# Composition, Predicted Functions and Co-occurrence Networks of Rhizobacterial Communities Impacting Flowering Desert Events in the Atacama Desert, Chile

**DOI:** 10.3389/fmicb.2020.00571

**Published:** 2020-04-08

**Authors:** Marcia Astorga-Eló, Qian Zhang, Giovanni Larama, Alexandra Stoll, Michael J. Sadowsky, Milko A. Jorquera

**Affiliations:** ^1^Programa de Doctorado en Ciencias de Recursos Naturales, Universidad de La Frontera, Temuco, Chile; ^2^Laboratorio de Ecología Microbiana Aplicada (EMALAB), Departamento de Ciencias Química y Recursos Naturales, Universidad de La Frontera, Temuco, Chile; ^3^BioTechnology Institute, University of Minnesota, Saint Paul, MN, United States; ^4^Centro de Estudios Avanzados en Zonas Áridas (CEAZA), La Serena, Chile; ^5^Department of Soil, Water, and Climate, University of Minnesota, Saint Paul, MN, United States; ^6^Department of Plant and Microbial Biology, University of Minnesota, Saint Paul, MN, United States; ^7^The Network for Extreme Environment Research (NEXER), Scientific and Biotechnological Bioresource Nucleus (BIOREN), Universidad de La Frontera, Temuco, Chile

**Keywords:** Atacama Desert, rhizobacterial community, *Cistanthe longiscapa*, flowering desert, high-throughput sequencing, co-occurrence network

## Abstract

Flowering desert (FD) events consist of the rapid flowering of a wide variety of native plants in the Atacama Desert of Chile, which is categorized as the driest desert in the world. While ephemeral plants are an integral part of the desert ecosystem, there is little knowledge on plant-microbe interactions that occur during FD events. Consequently, the overall goals of this present study were to investigate changes in the composition and potential functions of rhizobacterial community of *Cistanthe longiscapa* (*Montiaceae*) during the 2014 and 2015 FD events and determine the composition, potential functions, and co*-*occurrence networks of rhizobacterial community associated with the root zone of *C. longiscapa* during pre- (PF) and full-flowering (FF) phenological stages. Results of this study showed that the Proteobacteria and Actinobacteria were the dominant taxa in rhizosphere soils during the three FD events (2014, 2015, and 2017) examined. In general, greater microbial richness and diversity were observed in rhizosphere soils during the 2015-, compared with the 2014-FD event. Similarly, predicted functional analyses indicated that a larger number of sequences were assigned to information processing (e.g., ion channel, transporters and ribosome) and metabolism (e.g., lipids, nitrogen, and sulfur) during 2015 compared with 2014. Despite the lack of significant differences in diversity among PF and FF stages, the combined analysis of rhizobacterial community data, along with data concerning rhizosphere soil properties, evidenced differences among both phenological stages and suggested that sodium is a relevant abiotic factor shaping the rhizosphere. In general, no significant differences in predicted functions (most of them assigned to chemoheterotrophy, magnesium metabolisms, and fermentation) were observed among PF and FF. Co-occurrence analysis revealed the complex rhizobacterial interactions that occur in *C. longiscapa* during FD, highlighting to *Kouleothrixaceae* family as keystone taxa. Taken together this study shows that the composition and function of rhizobacteria vary among and during FD events, where some bacterial groups and their activity may influence the growth and flowering of native plants, and therefore, the ecology and trophic webs in Atacama Desert.

## Introduction

**FIGURE 1:**
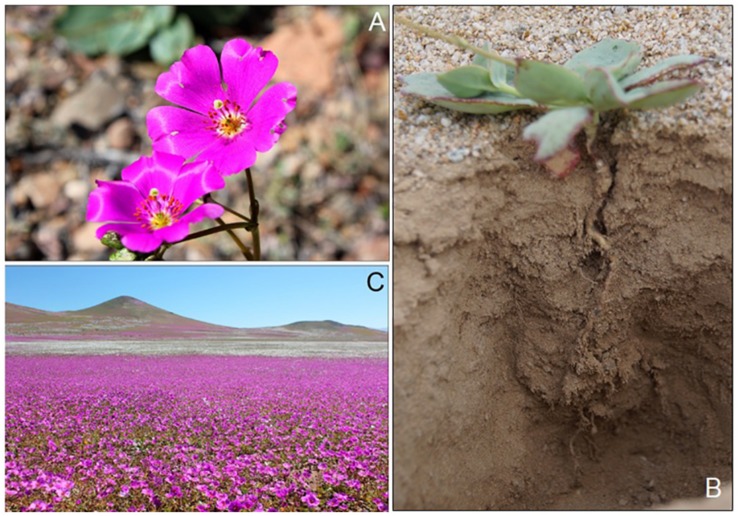
Flowers of *Cistanthe longiscapa* **(A)** and root in soil **(B)**. Mantles of *C. longiscapa* during full-flowering **(C)** stage at 2017 flowering desert event in Atacama Desert.

The Atacama Desert, located in northern Chile (from 18°24′S to 29°55′S), is considered as the driest non-polar place on Earth (Clarke, 2006). The Atacama Desert is characterized by a combination of subtropical climate of high pressure and a cold coastal Humbolt current that creates a constant temperature inversion, offshore winds, and shadow effect that restricts moisture advection from east (Pacific Ocean) to west (Los Andes mountains) (Houston and Hartley, 2003; Clarke, 2006). However, the Atacama Desert is periodically filled with life and color in a phenomenon called the “flowering desert (FD),” which corresponds to an explosive bloom of dormant desert plants produced by the presence of water as precipitation (Vidiella et al., 1999). During FD events (Figure 1), also named as “blooming deserts” (Chávez et al., 2019), productivity may be extremely high, supporting a rich but short-lived biotic assemblage. The flowering occurs preferentially on mantles (Figure 1C) of light wind-sands and, to a lesser extent, on the stony substrates of the plains that frame the sedimentary marine terraces, located between 100 and 300 m above sea level. The FD events have relevant ecological, social and economic implications for the Atacama region, activating trophic networks in the desert (e.g., herbivores and pollinators), which are resources for tourist activities and domestic livestock. The latter depends of this short period of high vegetal productivity (Gutiérrez, 2008). Despite the ecological importance of FD, the impact of microbial communities in growth and flowering of desert native plants remain unknown, particularly those related to plants and their roots.

While several studies have examined deserts around the globe for multiple inquiries (Beaulac et al., 2009), studies on FD events are still scarce because of the issues about their occurring prediction, determined in most of the cases for the water availability. In some deserts, the bloom of plants is an annually event (Cowling et al., 1999), while it is sporadically in others (Singh and Singh, 2011). Because of that, the main rhizobacteria associated to these ephemeral plants, and their role in the ecology of this events have been scarcely reported. However, some studies in Sonoran Desert and Saudi Arabia have reported the important role of the plant biotic interactions in shaping the species diversity (Yasir et al., 2015; Franklin et al., 2016). In Atacama Desert, *Cistanthe longiscapa* (Barneoud) Carolin ex Hershkovitz is a member of *Montiaceae* family, is an endemic annual herb, and it is one of the most representative groups of native plants during FD events (Stoll et al., 2017). Nonetheless, information concerning its physiology and association with microorganisms are extremely scarce. A recent study has reported that *C. longiscapa* microbiota significantly differed among rhizosphere soils and bulk soils in rainy year, but they are closer in dry year (Araya et al., 2019), suggesting differences in the ecological functions of microbial communities in each niche. Orlando et al. (2010), proposed that the bacterial community during FD bloom was likely involved in nitrogen (N) cycling. Moreover, plants and microbial communities have been shown to increase stabilization and contents of nutrients (N and carbon [C]) in desert soils (Orlando et al., 2012).

Recent high-throughput sequencing (HTS) studies have also described a wide diversity of bacteria associated with the rhizosphere (soil influenced by roots) of native plants in the Atacama Desert, including potential plant growth-promoting bacteria (Jorquera et al., 2014). However, there is a dearth of information concerning the diversity and role of rhizobacterial communities on the growth, survival, and fitness of FD plants, such as *C. longiscapa*. In this context, this study was designed in two stages: Firstly, we investigated the differences in composition and potential functions between rhizobacterial community of *C. longiscapa* during full-flowering (FF) stage in two consecutives FD events (2014 and 2015). Secondly, we investigated the composition, potential functions and co-occurrence networks of the rhizobacterial community associated with of *C. longiscapa* during the pre-flowering (PF) and FF phenological stages in a third FD event occurred on 2017, in order to elucidate the connections and key taxa in bacterial community during an unique FD event under two phenological stages.

## Methodology

### Stage 1: Composition and Predicted Functions of the Rhizobacterial Community During the 2014 and 2015 FD Events

#### Sampling

For this first stage of the investigation, rhizosphere soil samples were collected from three *C. longiscapa* mantles (about 50–70 m^2^ each), located in three different locations (27°28′03″S, 70°50′22″W; 28°22′07″S, 70°49′07″W; 28°46′10″S, 70°57′53″W) during the FF stages of local blooms in 2014 (October) and an extensive bloom in 2015 (September) FD events. The FF stage was defined as the period when >90% of the plant mantles were in full bloomed. In each year, five rhizosphere soil samples (∼20 g, from 6 to 10 plants) were randomly taken in a 20 m transect of each mantle to a depth of 5–10 cm excavating the soil with a sterile trowel and removing live superficial roots (1-2 mm in diameter), including the soil that adhered to the roots (Figure 1B). Rhizosphere soil was defined as that which adhered to the roots following shaking. The rhizosphere soil samples were deposited in sterile plastic bags, and immediately transported on ice to the Applied Microbial Ecology laboratory at La Frontera University. The rhizosphere soil samples were stored at −4°C until used for analysis.

#### Total DNA Extraction

For DNA extraction purposes, homogenized hizosphere soil samples (0.5–1 g) were pre-treated by vortexing for 1 h in 2 mL of sodium phosphate buffer (0.1 M, pH 8) and centrifuged at 16,000 × *g* for 10 min (He et al., 2005). The pellet was subjected to cell disruption by bead-beating for 30 s using a Powerlyzer^®^ homogenizer (Mo-Bio Laboratories, Carlsbad, CA, United States), and the solution was subjected to DNA purification using Power Soil^®^ DNA Isolation Kits (Mo-Bio Laboratories), according to manufacturer instructions. The quality and quantity of DNA extracts were measured using a microplate spectrophotometer (Multiskan GO, Thermo Fisher Scientific, Inc., Waltham, MA, United States). The DNA purity was assessed by determination of the A280/A260 absorbance ratios and only DNA extracts with absorbance ratios of ≥1.8 were used for bacterial community analyses.

#### High-Throughput DNA Sequencing

Purified DNA extracts from rhizosphere soil samples were initially submitted to Macrogen, Inc. (Seoul, South Korea) for 454-pyrosequencing (Roche) using 16S rRNA as a gene target. The selection of primer set and sequencing of 16S rRNA gene libraries were according to the Macrogen, Inc. protocol and recommended for Roche 454 GS-FLX System using Titanium Chemistry (454 Life Sciences). Briefly, 16S rRNA gene libraries were prepared by PCR using the universal bacterial primer UNI_AMP-27F (5′-Zxxx GAG TTT GAT CMT GGC TCA G-3′ and UNI_AMP-518R (5′-K WTT ACC GCG GCT GCT GG-3′) (Lee et al., 2010), where Z and K represent two pyrosequencing primers (CCATCT CAT CCC TGC GTG TCT CCG ACT CAG and CCTATC CCC TGTG TGC CTT GGC AGT CTC AG), and xxx was designed for the sample identification barcoding key. The PCR reaction was as follow: a hot start at 95°C for 3 min, PCR amplification was carried out for 35 cycles at 94°C for 15 s, 55°C for 45 s, and 72°C for 1 min. A final extension step was carried out at 72°C for 8 min. The 16S rRNA gene libraries were sequenced by with Roche 454 GS-FLX System using Titanium Chemistry (Roche Diagnostics Corporation, Life Sciences, Branford, CT, United States).

#### Analysis of Rhizobacterial Composition

The analysis of rhizobacterial community composition was done as described by Jorquera et al. (2016). Briefly, 454-pyrosequencing data was processed and analyzed using QIIME software, version 1.9.1 (Caporaso et al., 2010). Denoising, filtering low quality reads (phred score > 25), trimming of the barcode sequences and chimera check were carried out according to standard operating procedures for QIIME^1^. Raw sequencing data were deposited in the Sequence Read Archive (SRA) of NCBI under accession number SRR6461105.

Taxonomic assignment (the 97% level) was carried out using UCLUST algorithm^2^ and Greengenes Database Release 13_5 (DeSantis et al., 2006)^3^. The relative abundance of bacterial taxonomic groups was computed and visualized by using Geneious version 7^4^. Then, the richness (observed operational taxonomic units, *S*_*obs*_) and diversity (Shannon and Simpson indexes) of total bacterial communities was analyzed by using QIIME. In addition, the similarities between rhizobacterial communities were analyzed based on a distance matrix constructed using Bray-Curtis calculator and visualized as a non-metric multidimensional scaling (NMDS) plot using R software^5^.

#### Prediction of Rhizobacterial Community Functions

In order to obtain a first approximation of the metabolic potential of bacterial communities, a predictive functional analysis of rhizobacterial communities was performed by using PICRUSt software, version 1.1.0 (Langille et al., 2013). This analysis allows inference of the functional profile of a microbial community based on marker gene survey (16S rRNA gene), and we put attention in the differences among 2014 and 2015 FD events in some predicted functions with genetic (e.g., ribosomal content and tRNA biosynthesis), environmental information processing (e.g., signalling molecules and membrane transport) and metabolism (e.g., C and N metabolism) relevance. Metabolic profile inference was performed to predict KEGG Orthology (KO) and Clusters of Orthologs Groups (COG) classes via the PICRUST software. The Nearest Sequenced Taxon Index (NSTI) was used to express the expected uncertainty in the predictions. The resulting functional profiles were analyzed with the Tukey–Kramer *post-hoc* test and visualized using STAMP (Parks et al., 2014).

### Stage 2: Composition and Predicted Functions of Rhizobacterial Community During 2017 Flowering Desert Event

Considering the differences found in the composition and predicted functions of rhizobacterial communities among 2014 and 2015 FD events, a new sampling was done during the 2017 FD event, and focused in two different phenological stages (PF and FF) and adding interactive bacterial networks analysis. The PF stage was defined as the vegetative stage before the emergence of the floral buds, whereas FF stage was defined as >90% of the plant mantles were full bloomed.

#### Sampling

Rhizosphere soil samples was collected during PF (August) and FF (September) stages in 2017 FD event. In this sampling, PF was defined as the stage before the appearance of floral buds and plants in vegetative growth. Rhizosphere soil samples (∼20 g) were collected and processed as described above and taken from the same locations of *C. longiscapa* during 2014 and 2015 FD events. Rhizosphere soil samples (∼20 g) were subjected to chemical analysis as follow: soil pH was measured in 1:2.5 soil/deionized water suspensions. Available phosphorus (P_*Olsen*_) was extracted using 0.5 M Na-bicarbonate and analyzed using the molybdate blue method (Murphy and Riley, 1962). Organic matter was estimated by the Walkley–Black method (Combs and Nathan, 1998). Exchangeable cations of potassium (K^+^), calcium (Ca^2+^), magnesium (Mg^2+^), and sodium (Na^+^) were extracted with 1 M ammonium acetate, pH 7.0, and analyzed by flame atomic adsorption spectrophotometry (FAAS) (Warncke and Brown, 1998). The rhizosphere soil properties are shown in Table 1.

**TABLE 1:** Chemical properties of rhizosphere soils from *Cistanthe longiscapa* during 2017 flowering desert event.

**Samples**	**PF**	**FF**
P_*Olsen*_ (mg kg^–1^)	21.2	20.9
Organic matter (%)	1.51	1.54
pH_H__2__*O*_	8.6	8.2
K (cmol_(__+__)_ kg^–1^)	1	0.85
Na (cmol_(__+__)_ kg^–1^)	0.61	0.62
Ca (cmol_(__+__)_ kg^–1^)	75	81
Mg (cmol_(__+__)_ kg^–1^)	2	1.85
CEC^*a*^ (cmol_(__+__)_ kg^–1^)	78.61	84.32

*PF, pre-flowering stage; FF, full-flowering stage. ^*a*^CEC = cation exchange capacity = Σ(K, Ca, Mg, and Na).*

#### Total DNA Extraction and High-Throughput DNA Sequencing

Total DNA was extracted from rhizosphere soil samples by using Power Soil^®^ DNA Isolation Kits (Mo-Bio Laboratories) according to manufacturer instructions as described above. Purified DNA extracts from rhizosphere soil samples were then submitted at Macrogen, Inc. (Seoul, South Korea) for sequencing using 16S rRNA as gene target. It is necessary to mention that Macrogen, Inc. migrated from 454-pyrosequencing to Illumina technology for sequencing. The selection of primer set and sequencing of 16S rRNA gene libraries were according to the Macrogen, Inc. protocol and recommended for Illumina MiSeq platform (Illumina, Inc., San Diego, CA, United States). Briefly, 16S rRNA gene libraries were prepared by PCR using the universal bacterial primers 27F-AGA GTT TGA TCM TGG CTC AG and 1492R-TAC GGY TAC CTT GTT ACG ACT T. The PCR reaction was as follow: a start at 96°C for 1 min, PCR amplification was carried out for 25 cycles at 96°C for 10 s, 50°C for 55 s, and 60°C for 4 min. A final step was carried out at 15°C for until finished reaction.

#### Composition and Predicted Functions of Rhizobacterial Community

Sequence data was analyzed by using Mothur ver. 1.34.0 (Schloss et al., 2009). In brief, after trimming the low-quality regions at the ends of reads, reads were merged by using Fastq-join software (Aronesty, 2013), maintaining an average quality score >33. After removing the primer sequences, high quality sequencing reads were aligned on the basis of the Greengenes ver.13.8 database (McDonald et al., 2012). The UCHIME software was used to identify and remove probable chimeric sequences (Edgar et al., 2011). Raw sequencing data were deposited in the SRA of NCBI under accession number from SRR9329822 to SRR9329839.

For statistical analysis, the Mothur program was also used to calculate alpha diversity indices, Shannon index and the abundance-based coverage estimate (ACE), in samples. A distance matrix constructed using Bray-Curtis calculator were used to analyze similarities between bacterial communities and visualized as a NMDS plot using the R software^6^. The VennDiagram package in R was used to identify shared OTUs of bacterial communities (Chen and Boutros, 2011) between different phenological stages of *C. longiscapa*.

Finally, to predict the potential function of microbial community among PF and FF stages, the FAPROTAX program was used to obtain the functional table through the default settings based on taxonomic information found in Antarctic vascular plant (Louca et al., 2016).

#### Co-occurrence Network of Rhizobacterial Community During 2017 Flowering Desert Event

The co-occurrence network was generated through the WGCNA package based on Spearman correlation matrix as described by Ma et al. (2016). The OTUs were represented by the nodes and the correlations between OTUs were described as the edges in the topological graph, respectively. During the network construction, the appropriate similarity (0.807) was chosen according to random matrix theory (RMT) (Luo et al., 2006). In addition, the Benjamini and Hochberg false discovery rate (FDR) was used to adjust the *P* values and set up the threshold value 0.05 (Benjamini et al., 2006). The topological network properties were calculated via *igraph* package (Csardi and Nepusz, 2006). Likewise, the keystone taxa were determined on the basis of thresholds: OTUs with degree higher 8, closeness centrality greater than 0.15, and betweenness centrality smaller than 0.08 (Berry and Widder, 2014). Gephi software was used to visualize the network image (Bastian et al., 2009).

## Results

### Composition and Predicted Functions of Bacterial Community During 2014 and 2015 Flowering Desert Events

**FIGURE 2:**
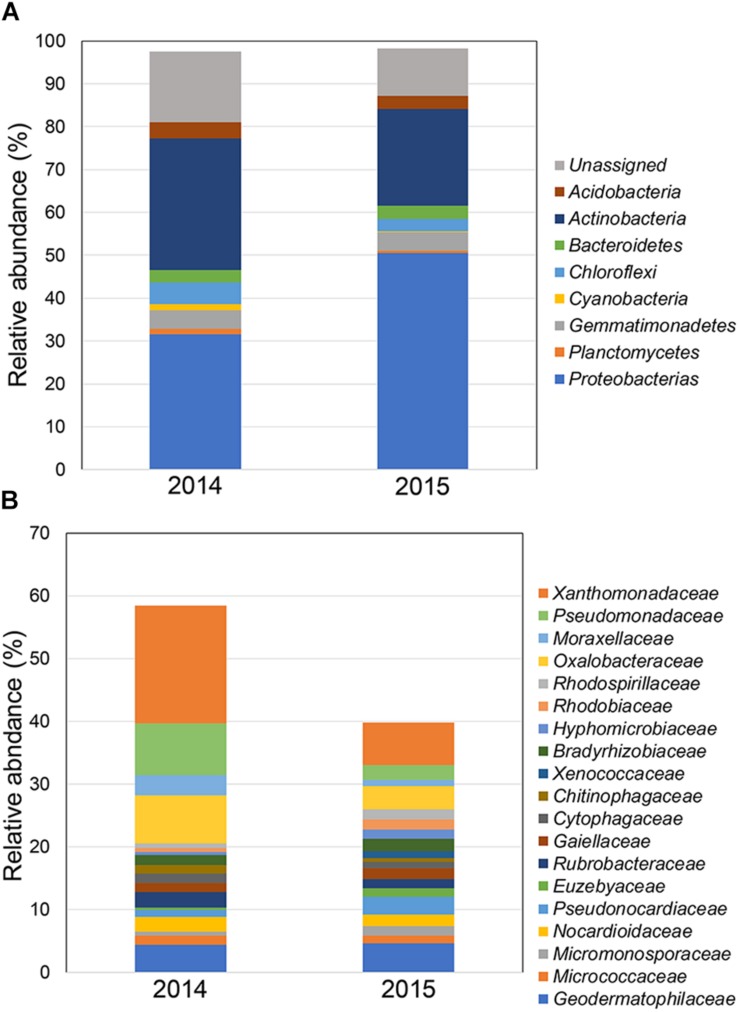
Average of relative abundance at phylum **(A)** and family **(B)** taxonomic levels in the total rhizobacterial community associated with *Cisthante longiscapa* during full-flowering stage of 2014 and 2015 flowering desert events in Atacama Desert.

**FIGURE 3:**
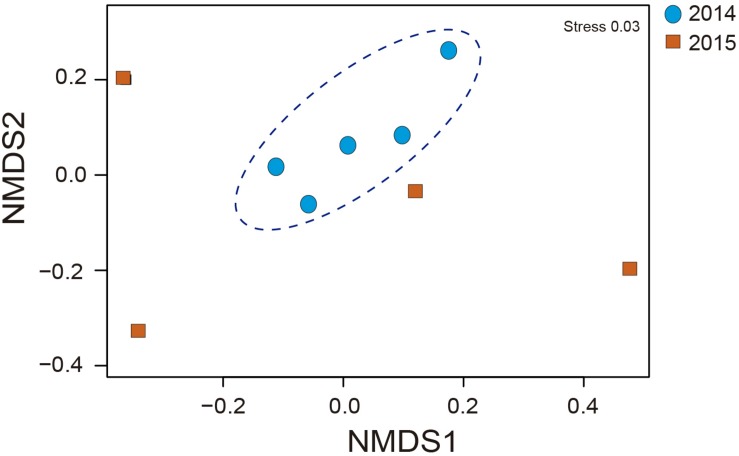
Nonmetric multidimensional scaling (NMDS) analyses derived from taxonomic data analysis of total rhizobacterial community associated with *Cisthante longiscapa* during full-flowering stage of 2014 and 2015 flowering desert events in Atacama Desert.

The relative abundances of the bacterial community in rhizosphere soil from samples obtained during 2014 and 2015 FD events are shown in Figure 2. At the phylum level (Figure 2A), rhizosphere soil samples showed that Proteobacteria and Actinobacteria were the dominant phyla in 2014 and 2015, with values ranging from 31.6 to 51.0% and from 30.6 to 22.7%, respectively. The next abundant phyla in rhizosphere samples were Chloroflexi and Gemmatimonadetes, with values ranging from 5.2 to 2.9% and from 4.3 to 4.2% during 2014 and 2015, respectively. Other phyla found among 2014 and 2015 samples were Bacteroidetes (2.9–3.0%), Acidobacteria (3.1–3.8%), Cyanobacteria (0.1–1.4%), and Planctomycetes (0.7–1.3%). At the family level (Figure 2B), rhizosphere soil samples showed a higher abundance of *Xanthomonadaceae* (18.74%), followed by *Pseudomonas* (8.26%), *Oxalobacteraceae* (7.70%), *Geodermatophilaceae* (4.36%), and *Rubrobacteraceae* (2.50%) during 2014. Similarly, *Xanthomonadaceae* (6.78%) was the dominant family found in 2015, followed by the *Geodermatophilaceae* (4.68%), *Oxalobacteraceae* (3.68%), *Pseudomonas* (2.40%), and *Rubrobacteraceae* (1.55%). Bacterial community coverage ranged from 96 to 97% with a significant (*P* ≤ 0.05) highest observed OTUs during 2015 respect to 2014 (Table 2). Significant higher (*P* ≤ 0.05) values of diversity indexes (Shannon and Simpsons) were observed in 2015 compared with 2014. This difference between 2014 and 2015 FD events was confirmed by NMDS, where rhizobacterial communities were more similar in 2014 than 2015 (Figure 3).

**TABLE 2:** Coverage and alpha diversity among bacterial communities in the rhizosphere of *Cistanthe longiscapa* during full-flowering in 2014 and 2015 flowering desert events.

**Year**	**Sample number**	**Coverage (%)**	***S*_*obs*_^†^**	**Shannon index**	**Simpson (D’)**
2014	5	97.25 ± 1.39^A,^*,^‡^	1,665.00 ± 496.39^B^	8.84 ± 1.36^B^	0.9876 ± 0.012^B^
2015	4	96.00 ± 1.25^A^	2,178.00 ± 179.61^A^	10.19 ± 0.2^A^	0.9973 ± 0.001^A^

^†^*S*_*obs*_: number of OTUs observed at 97% similarity. ^‡^Values represent mean ± standard error. *Different letter denotes statistical difference (*P* ≤ 0.05, Tukey test) in the same column.

**FIGURE 4:**
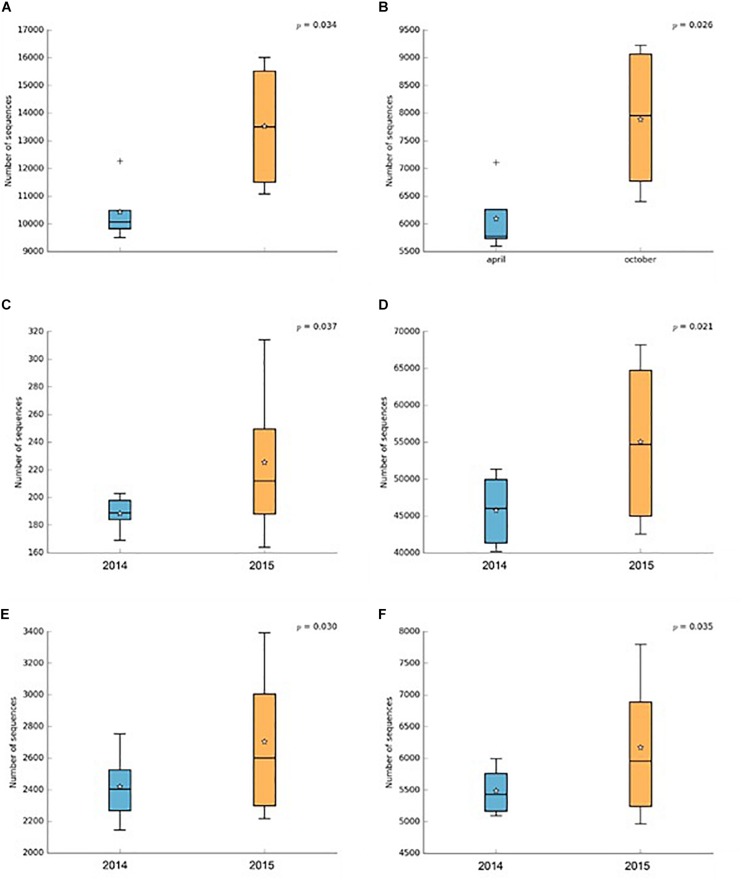
Number of sequences assigned to genetic information [ribosome **(A)** and aminoacyl-tRNA biosynthesis **(B)**], environmental [ion channels **(C)** and transporters **(D)**] and energy metabolisms [sulfur **(E)** and nitrogen metabolism **(F)**] in the total rhizobacterial community associated with *Cisthante longiscapa* during full-flowering stage of 2014 and 2015 flowering desert events in Atacama Desert.

In addition, PICRUSt software allowed inference of differences in some genetic, environmental and metabolic functions among 2014 and 2015 events (Figure 4). Thus, significant (*P* ≤ 0.05) higher number of sequences were assigned to genetic information processing, such as ribosome protein expression and aminoacyl-tRNA biosynthesis, during 2015 in relation to 2014 (Figures 4A,B). A significantly (*P* ≤ 0.05) higher number of sequences were also assigned to environmental information processing, such as ion channels and transporters, during 2015 compared to 2014 (Figures 4C,D). Similarly, a significantly (*P* ≤ 0.05) higher number of sequences were assigned to energy metabolism (sulphur [S] and N metabolism) during 2015 in relation to 2014 (Figures 4E,F).

### Composition and Predicted Functions of the Bacterial Community During 2017 Flowering Desert Event

**FIGURE 5:**
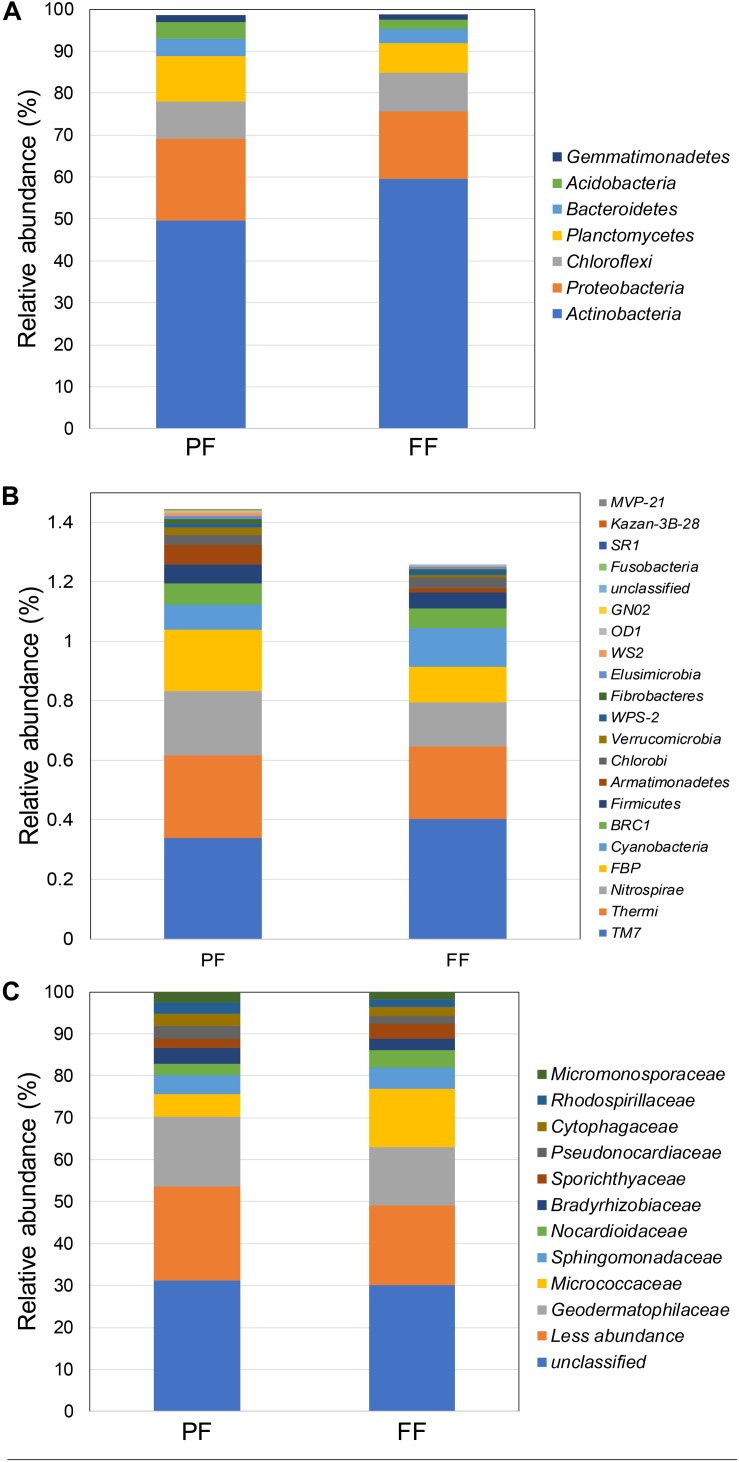
Average of relative abundance at phylum [major **(A)** and minor **(B)** abundance] and family **(C)** taxonomic levels in the total rhizobacterial community associated with *Cisthante longiscapa* during pre- (PF) and full-flowering (FF) stages of 2017 flowering desert event in Atacama Desert.

The relative abundances of total bacterial community in rhizosphere soil sampled during the PF and FF 2017 FD events are shown in Figure 5. As was found with 2014 and 2015 FD events, Actinobacteria and Proteobacteria were the predominant phyla in PF and FF rhizosphere soil during 2017 (Figure 5A). However, significantly (*P* ≤ 0.05) higher relative abundance of Actinobacteria was observed in FF (59.5%) than PF (49.6%) and yet significant smaller Proteobacteria were observed in FF (16.1%) compared with PF (19.5%). In terms of abundances, the Actinobacteria and Proteobacteria were followed by the Planctomycetes and Chloroflexi as dominant phyla, with values of 10 and 6, and 8 and 8% during PF and FF, respectively. Similar to previous FD events analyzed, other phyla found among the PF and FF were Bacteroidetes (4.2–3.5%), Acidobacteria (3.9–2.2%), and Gemmatimonadetes (1.6–1.3%%). Interestingly, greater abundances of these three phyla were observed in the PF than in FF, although there was not significantly different (ANOVA, *P* ≤ 0.05).

With respect to minor taxa (<1%), a higher abundance of bacterial groups was observed in PF compared with FF (Figure 5B); mainly highlighting the Cyanobacteria (0.08–0.13%), WS2 (0.008–0.004%), Nitrospirae (0.21–0.15%) and FBP (0.21–0.12%) phyla in both phenological stages of plants, respectively. At the family level (Figure 5C), a higher abundance of *Bradyrhizobiaceae* (2.8%) and *Pseudonocardiaceae* (2.1%) were found under PF compared with FF (2 and 1.7% respectively), contrasting with the relative abundance of *Micrococcaceae* (5.8%), *Nocardioidaceae* (3%) and *Sporichthyaceae* (1.8%) which showed higher abundance in FF (14, 4, and 2.5%, respectively).

**FIGURE 6:**
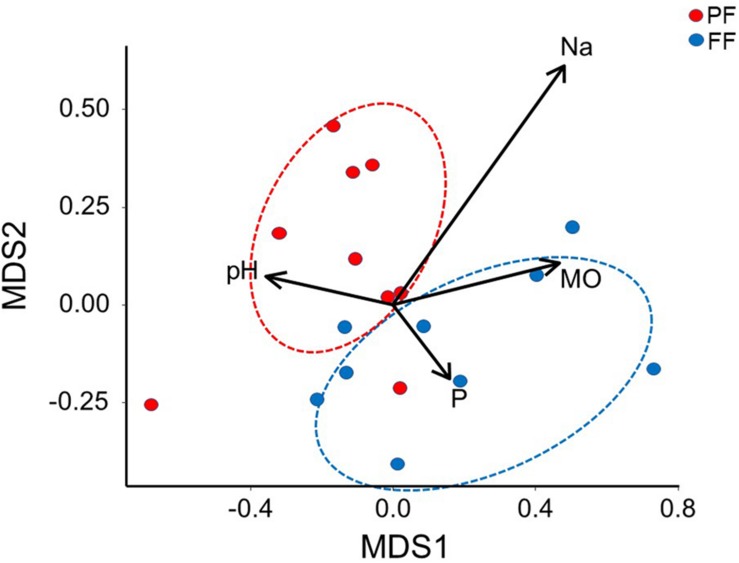
Non-metric multidimensional scaling (NMDS) analyses derived from taxonomic data of total rhizobacterial community and rhizosphere soil properties taken from *Cisthante longiscapa* during pre- (PF) and full-flowering (FF) stages of 2017 flowering desert event in Atacama Desert. O.M., organic matter; P_*Olsen*_, available phosphorus; Na, natrium.

In relation to the bacterial communities, NMDS analysis showed two significantly different groups during PF compared with FF (ANOSIM, *P* ≤ 0.05) (Figure 6). Interestingly, Na content in rhizosphere soils was the key parameter regulating bacterial communities in both PF and PP (*r*^2^ = 0.60, *P* = 0.01). Comparing the OTUs amount, only 28.0% (724 OTUs) and 23.3% (603 OTUs) were unique to PF and FF, respectively (Figure S1 Venn diagram). About 2,585 OTUs were shared among both stages (Supplementary Figure S1 Venn diagram). In addition, the percentage of coverage, the number of OTUs observed (97% similarity), and ACE were not significantly different between PF and FF stages (Table 3). In contrast, Shannon index was significantly greater (*P* = 0.05) in PF compared with FF.

**TABLE 3:** Coverage and alpha diversity among bacterial communities in the rhizosphere of *Cistanthe longiscapa* in 2017 flowering desert event at pre-flowering and full-flowering stages.

**Stage**	**Sample number**	**Coverage (%)**	***S*_*obs*_^†^**	**Shannon index**	**ACE^‡^**
Pre-flowering	9	98.99 ± 0.07*,^A,^**	1,533 ± 79^A^	5.85 ± 0.1^A^	1,851 ± 102^A^
Full-flowering	9	99.16 ± 0.07^A^	1,298 ± 79^A^	5.52 ± 0.1^B^	1,565 ± 102^A^

^†^*S*_*obs*_: number of OTUs observed at 97% similarity. ^‡^ACE: abundance-based coverage estimates. *The values represent least squares mean ± standard deviation from *n* = 9. **Sample groups sharing the same letter in each column did not vary significantly (*P* ≤ 0.05; Bonferroni test).

**FIGURE 7:**
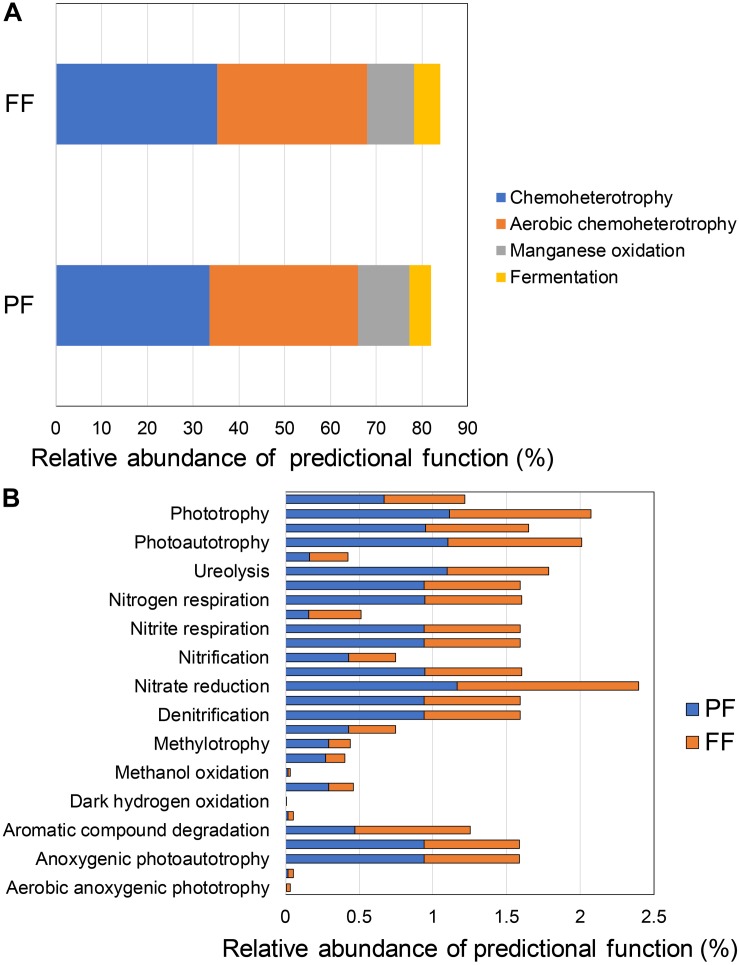
Average of relative abundance of major **(A)** and **(B)** minor functions predicted in the total rhizobacterial community associated with *Cisthante longiscapa* during pre- (PF) and full-flowering (FF) stages of 2017 flowering desert event in Atacama Desert.

The FAPROTAX analysis predicted similar major functions among PF and FF, where major abundances were attributed to chemoheterotrophy (33.6–35.2%), aerobic chemoheterotrophy (32.3–32.8%), manganese oxidation (11.3–10.3%), and fermentation (4.6–5.6%) (Figure 7A). When minor predicted functions were taken into account, there were greater phototrophy (1.12–0.96%), nitrate reduction (1.16–1.23%), photoautotrophy (1.10–0.91%) and ureolysis (1.10–0.69%) in in PF and FF (Figure 7B). In a more detailed analysis, functions attributed to oxygenic photoautothrophy, N fixation and aromatic compounds degradation showed lower abundance in PF (0.16, 0.16, and 0.47%, respectively) compared with FF (0.26, 0.35, and 0.78%, respectively). It is particularly noteworthy that functions attributed to methane oxidation, dark hydrogen oxidation, aromatic hydrocarbon degradation, aliphatic non- methane hydrocarbon degradation, and aerobic anoxigenic phototrohpy presented the lowest abundances.

### Co-occurrence Network of Bacterial Community During 2017 Flowering Desert Event

**FIGURE 8:**
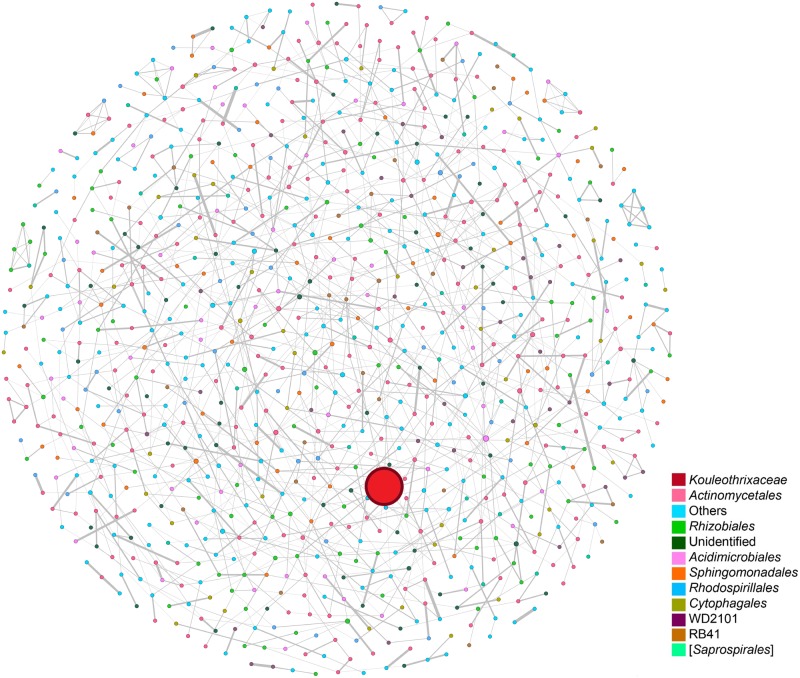
Co-occurrence network analysis of the total rhizobacterial community associated with *Cisthante longiscapa* during pre-(PF) and full-flowering (FF) stages of the 2017 flowering desert event at the Atacama Desert. The size of each node (representing OTUs) is proportional to the number of connections (degrees). The size of edges connecting nodes represent both strong (spearman’s ρ > 0.807) and significant (*P* ≤ 0.05) correlations between OTUs. Node colors represent the taxa indicated.

The co-occurrence network of the rhizobacterial community of *C. longiscapa* included 970 nodes (e.g., OTUs) and 1,324 edges, indicative of the association between OTUs (Table 4). The average network diameter, modularity index, and transitivity were 17, 0.809, and 0.19, respectively (Figure 8). The results also show one keystone taxon, as the “engineering driver” leading the difference in the complex rhizosphere network between the two scenarios. This taxon (OTU) was classified into *Kouleothrixaceae* at the family level. Despite its importance, the abundance of this keystone taxon was only 0.02% in FF and was not detected in PF.

**TABLE 4:** Co-occurrence Network properties of the rhizobacterial communities associated with *Cisthante longiscapa* during pre- and full-flowering stages of 2017 flowering desert event in Atacama Desert.

**Parameter**	**Value**
Vertex number	970
Edge number	1.324
Diameter	17.438
Average path length	8.873
Average nearest neighbor degree	3.154
Betweenness centralization	0.059
Closeness centralization	0.002
Density	0.002
Degree centralization	0.006
Degree assortativity	0.104
Transitivity	0.193

## Discussion

Results of this current study showed that rhizobacterial community compositions associated with *C. longiscapa* during 2014 and 2015 FD events were determined by the presence of *Proteobacteria* and *Actinobacteria* as main phyla. Similarly, *Proteobacteria* and *Actinobacteria* have previously been reported as dominant phyla in the rhizosphere of native plants (*Atriplex* sp. and *Stipa* sp.) in Atacama Desert by using denaturing gradient gel electrophoresis (DGGE) and 454-pyrosequencing of 16S rRNA genes (Jorquera et al., 2014, 2016). By using Illumina MiSeq sequencing of the 16S rRNA gene, rhizobacterial communities associated with Atacama Desert high altitude native plants (*Calamagrostis crispa*, *Nassella nardoides*, *Jarava frigida*, and *Pycnophyllum bryoides*) were dominated by *Proteobacteria*, whereas *Actinobacteria* was dominant in bulk soils (Fernández-Gómez et al., 2019). In FD events during 2005 and 2006, Actinobacteria, Proteobacteria, Firmicutes, and Bacteroidetes were found as dominant phyla by using cloning and sequencing of the 16S rRNA and ammonia monooxygenase (*amo*A) genes (Orlando et al., 2010).

Here we show that the Proteobacteria and Actinobacteria were followed by Chloroflexi and Gemmatimonadetes as dominant phyla. Despite the differences in relative abundances, Chloroflexi seems to be present in most of the soils around the including the rhizosphere microbiomes (Mendes et al., 2011). Members of Chloroflexi has also been found in desert soil, including various soil communities and hypoliths of the Atacama Desert (Neilson et al., 2012). With respect to Gemmatimonadetes, it is known that this bacterial group can thrive in hyperarid soils with extremely low organic C and N levels, suggesting that their abundance in arid soils implies that they are important colonists (DeBruyn et al., 2011). Gemmatimonadetes have also been described as common inhabitants of rhizosphere soils in Chilean ecosystems (Jorquera et al., 2016), including FD event (Orlando et al., 2010). Other phyla found as component of *C. longiscapa* rhizosphere included Bacteroidetes, Acidobacteria, Cyanobacteria, and Planctomycetes. These taxa have been found in arid and semiarid regions of the world, including cold deserts in the Antarctic (Aislabie et al., 2006), hot deserts in Chile (Navarro-González et al., 2003), and undisturbed and agro-ecosystems (Jorquera et al., 2014; Tian et al., 2015).

At family the level, *Xanthomonadaceae* was the principal bacterial family detected in the 2014 FD event, but with a lower abundance than in the 2015 FD event. *Xanthomonadaceae* family is recognized as typical soil bacteria (Assis et al., 2017) and members of this taxa can be found in several environments, including rhizosphere soils (Pétriacq et al., 2017). Other families, such as *Oxalobacteraceae*, *Geodermatophilaceae*, and *Rubrobacteraceae*, were also found in both FD events, but in different proportions. Moreover, *Pseudomonas*, *Oxalobacteraceae*, and *Rubrobacteraceae* diminished in 2015 compared to 2014, and *Geodermatophilaceae* was found in the same proportion for both FD events. The family *Geodermatophilaceae* harbors many strains adapted to extreme ecological niches, such as the desert core in the Atacama Desert (Castro et al., 2018), and at high altitudes and in hyper-arid soils of the Chilean Central-Andes (Bull et al., 2018). Coincidently with 2014 and 2015 FD events, 2017 FD event showed Proteobacteria and Actinobacteria, followed by Chloroflexi and Planctomycetes as dominant phyla in both PF and FF stages.

In relation to rhizobacterial diversity, alpha diversity measured by the Shannon index (H’) showed significant differences among 2014 and 2015 FD events, with values of 8 and 10, respectively. Similar values were obtained in the rhizosphere of invasive plants by Cheng et al. (2019), which rapidly grow and are in constant expansion. The Simpson index, as well as H’, corroborated the differences in rhizobacterial diversity for both FD events, with values lower (0.98 and 0.99) to those found in rhizosphere of desert plants (2.3–3.9) (Aguirre-Garrido et al., 2012) and comparable to those obtained for plants with agricultural importance (0.87–0.97) (Yang et al., 2016).

Differences in rhizobacterial communities among 2015 and 2014 FD events were also confirmed by NMDS analysis derived from taxonomic data. A higher-level grouping in the rhizobacterial communities was evidenced during 2014 compared with 2015 FD, which suggests that rhizobacteria bacterial communities had similar composition and maybe exert similar functions in rhizosphere during flowering of *C. longiscapa.* Shifts in functions of bacterial community might not be coincident with changes in taxonomic as observed by Jorquera et al. (2014) and Marileo et al. (2016) using phylogenetic (16S rRNA and *rpo*B) and functional (*amo*A, *nif*H, and APase) genes. The recruitment of specific microbial community by *C. longiscapa* seems to contribute a optimal plant development under unique Atacama Desert conditions (Araya et al., 2019). In this context, rhizobacterial community functions predicted by PICRUSt shows significant differences (*P* ≤ 0.05) in numbers of sequences involved in environmental and genetic information processing among 2014 and 2015 FD events. A greater number of sequences were assigned to ion channels, transporters, ribosome and aminoacyl-tRNA biosynthesis in 2015, compared with 2014. Ion channels and transporters are formed by proteins and are responsible to regulate ion transportation (Na^+^, K^+^, Ca^2+^, and Cl^–^) and molecules (ethanol, urea, amino acids, glucose 6-phosphate, among others) through cellular membranes maintaining electrochemical gradient and volume of bacterial cells (Potts, 1994). Transport of ions is crucial to prevent cellular damage caused by dehydration or desiccation, especially in hot and desert environments, where the amount of salts and ions in soils, compared with intracellular composition in bacteria, is higher favoring the loss of water in bacterial cell (Rothschild and Mancinelli, 2001; Zhang and Yan, 2012). Differences in energy and metabolism of S and N during 2014 and 2015 FD events were also predicted. Higher number of assigned sequences related to the abovementioned functions were observed during 2015, compared with 2014 FD event. Similarly, to ribosomes and aminoacyl-tRNA biosynthesis, S and N metabolisms are involved in proteins synthesis. N metabolism is also responsible of essential processes in the rhizosphere such as N fixation, nitrate reduction, denitrification, nitrification, etc. (Klemme, 1989; Richardson and Watmough, 1999). In addition, microbial colonization is the predominant form of primary productivity and N input in such extreme desert regions (Lacap et al., 2011). Interestingly, even though two different platforms for HTS were used for rhizobacterial community analyses in this research, both provide a comparable view of the community sampled, regardless of differences in read length and sequencing technologies, as demonstrated by Luo et al. (2012). Regarding PF and FF, some significant shifts in the *Proteobacteria* and *Actinobacteria* abundance were observed. Actinobacteria increased in their relative abundance in FF while Proteobacteria decreased. Chloroflexi had a similar relative abundance in both stages and Planctomycetes was less abundant in FF than PF. These changes in the composition of total rhizobacterial communities during PF and FF may be explained by the metabolic differences according to plant phenological stages (Houlden et al., 2008). Some investigations observed changes not only in the structure but also in the functionality of culturable rhizobacterial communities during different phenological stages of plants (Ruiz-Palomino et al., 2005). In *C. longiscapa* rhizosphere, assignations of *amo*A gene to different *Nitrospira* clusters were observed before, during and after FD events (Orlando et al., 2010). In addition, it is commonly reported that an increase in the secretion of proteins, organic acids, and phenolic compounds by plant roots during the flowering stage (Lucas García et al., 2001), inducing a higher growth and activity of bacteria as well as the recruitment of specific bacterial groups in the rhizosphere (Lundberg et al., 2012; Edwards et al., 2015).

At the family level, the dominant groups were the *Bradyrhizobiaceae, Pseudonocardiaceae, Micrococcaceae, Nocardioidaceae*, *Sporichthyaceae*, at different proportions in both PF and FF stages during 2017 FD event. In the case of *Bradyrhizobiaceae* and *Pseudonocardiaceae*, both families were present in less abundance in PF than in PF. *Bradyrhizobiaceae* is known for the N fixation capacities of their representatives as one of the most important ecological properties with potential application in agriculture besides other diazotrophic members into the *Alphaproteobacteria* class (Marcondes de Souza et al., 2014). In relation to *Pseudonocardiaceae*, some of their representatives are able to produce bioactive compounds with antimicrobial activity, and to thrive under strongly UV-B irradiation (Bull et al., 2018). Thus, the aforementioned rhizobacterial families could be important collaborators for the establishment of a plant cover in the desert soil and their shift in abundance may be explained precisely for these features. In contrast, families *Micrococcaceae, Nocardioidacea*, and *Sporichthyaceae* increased in abundance in PF. These abundance changes could also be associated with the principal features of each family where *Micrococcaceae* and *Nocardioidaceae* harbor representatives considered the most abundant degraders of plant residue and primary degraders of organic material in crop lands (Ortiz-Cornejo et al., 2017) and have a significant role in degradation processes and nutrient cycling (Tóth and Borsodi, 2014). This degrading capacity would allow to these rhizobacterial groups to be more abundant in FP than FF. Interestingly, the *Sporichthyaceae* have been described as solar radiation tolerant microbes and found in impoverished soils (Bull et al., 2018). However, *Sporichthyaceae* is one of the less studied bacterial group mostly owing to their slow growth rate and requirements (Normand, 2006).

In relation to the diversity of rhizobacterial community among the FF and PF, no differences were observed in both phenological stages, except with Shannon index, which is consistent with the explanation given by Araya et al. (2019) who indicate that a small group of bacteria is consistently associated with *C. longiscapa* rhizosphere. However, differences among phenological stages were evidenced when rhizobacterial communities were evaluated with some rhizosphere soils properties using NMDS analysis. The grouping pattern by NMDS is consistent with the greater supply of nutrients for bacteria during FF (secreted by the plant, and the recycling of plant detritus), which modulates the variety of bacteria inhabiting this niche (Miki et al., 2010). In contrast, during PF, when fewer resources are available, a higher competence between bacterial groups would occur, hence the relative abundances at phyla level could vary drastically among phenological stages. NMDS also showed sodium (Na) as the main abiotic factor influencing the rhizobacteria grouping, more than organic matter (O.M.), available phosphorus (P_*Olsen*_) or pH. Atacama Desert soils are characterized by their high salinity where commonly are isolated salt tolerance culturable bacteria (4–8% NaCl) (Okoro et al., 2009; Maza et al., 2019). Factors influencing the rapid variation and adaptation of rhizobacterial communities are identified as plant genotype, phenological stages of plants, composition of rhizodeposits, bacterial assemblages, microenvironment generated by a whole set of plants or bacterial interactions (Fernández-Gómez et al., 2019), especially considering that some bacterial taxa observed in this study are also influenced in their distribution and diversity in short periods of time by diurnal cycling (Staley et al., 2017).

Analysis of predicted functions in rhizobacterial communities showed that the greatest amount of sequences was assigned to chemoheterotrophy, aerobic chemoheterotrophy, manganese oxidation, and fermentation in both phenological stages. These metabolic processes are related to energy production from different metabolic sources, explained by C-containing primary and secondary metabolites from the root exudates (Bais et al., 2006), low- and high-molecular weight compounds, proteins, organic acids, sugars and some polysaccharides (mucilage), which must be metabolized by rhizobacteria in order to obtain energy for microbial survival (Walker et al., 2003; Ladwig et al., 2015). In addition, minor functional predictions were mainly assigned to different metabolic processes, such as photoautothropy and N metabolism (e.g., nitrate reduction and ureolysis), processes related to N fixation and N transformation in soil (Oshiki et al., 2018).

The co-occurrence network of the rhizobacterial community of *C. longiscapa* revealed the presence of 970 nodes and 1,324 edges. These results were higher to other rhizosphere analyses in desert plants, were nodes range from 375 to 488 (Gunnigle et al., 2017). In general, these values are similar to those found in rhizosphere of some Mediterranean plants with values ranging from 350 to 1,000 nodes (Shi et al., 2016). In the same study, the amount of edges for *Avena fatua* was lower (40–1,200 edges) than the amount obtained for *C. longiscapa*, during the 2017 FD event, indicating more connections between different bacterial taxa. In addition, the modularity index was 0.809, similar to those obtained for plants growing under desert environments affected for monsoon climate (Zheng et al., 2017) indicating that the network has a modular structure (Newman, 2006), with groups of highly connected nodes within the group and few connections outside the group (Barberán et al., 2012). The networks also showed to members of *Kouleothrixaceae* family (*Chloroflexi* phylum) as keystone taxon in the *C. longiscapa* rhizosphere, despite that its abundance was only 0.02% in FF and did not detected in PF. In other environments (such as sludge), *Kouleothrixaceae* representatives have been characterized as bacteria specialized in polysaccharide degradation produced by other microorganisms and on decaying cells (Kragelund et al., 2007), which is an important feature for bacteria in arid environments, particularly in rhizosphere, where C sources are limited to plant exudates, EPS produced for microorganisms and plant debris. Members of the family *Kouleothrixaceae* have been reported in the rhizosphere of Nickel (Ni)-hyperaccumulator plants, but there is no correlation between the relative abundance of *Kouleothrixaceae* and metals (Niquel), cations (Cadmium) and O.M. was found (Lopez et al., 2017). Despite the low abundance of members of the *Kouleothrixaceae* family, they are likely still important for ecosystem function. Several studies currently highlight the relevance of low abundance and rare taxa in nature, indicating population dynamics, dispersion, predation and persistence of these underrepresented bacteria (Lynch and Neufel, 2015). Moreover, some studies exploring the microbial diversity from Atacama Desert soil, have reported that rare taxa are able to contribute in the dynamic (Shade et al., 2014) and resilience (Idris et al., 2017) of the total soil bacterial community acting as a reservoir that can rapidly respond to environmental changes.

## Conclusion

The flora and fauna living in Atacama Desert are highly adapted to local harsh conditions, where FD events and their associated bacterial communities are pivotal for the ecology, tourism and domestic livestock production of the Atacama region. In this study, the analysis of the bacterial communities revealed that *Proteobacteria* and *Actinobacteria* phyla are the dominant taxa in the *C. longiscapa* rhizosphere among and during (FD) events. However, significant differences in the composition of total rhizobacterial communities were revealed not only among the 2014 and 2015 events but also among pre- (PF) and full flowering (FF) stages during 2017 FD event. Similarly, higher number of predicted functions (information processing and metabolism) were assigned to 2015 compared with 2014 FD event, but no big differences in predicted functions were found among PF and FF stages during 2017 FD event, where chemoheterotrophy, manganese oxidation and fermentation represented the major assignations. The co-occurrence network analysis also revealed the complex bacterial association in *C. longiscapa* rhizosphere during FD events, highlighting *Kouleothrixaceae* family as key stone taxa with higher number connections within community, but with a low abundance (0.02%). Our results not only reveal the compositions and potential functions of bacterial communities but also the relevance of minor taxa (or rare taxa) impacting rhizosphere processes for fast growth of native plants during FD, which is one of the most extraordinary (and scarcely studied) natural event in Atacama Desert, one of the driest places in the globe.

## Data Availability Statement

The datasets generated for this study can be found in the SRA of NCBI under accession numbers SRR6461105 and SRR9329822 to SRR9329839.

## Author Contributions

MA-E, QZ, and MJ designed the research. AS provided the 2014 and 2015 samples. MA-E, QZ, and GL performed the laboratory work and data analysis. MA-E, QZ, MS, and MJ wrote the manuscript and designed the tables and figures. All authors revised the manuscript and approved the final version.

## Conflict of Interest

The authors declare that the research was conducted in the absence of any commercial or financial relationships that could be construed as a potential conflict of interest.

## Footnotes

1 http://qiime.org/tutorials/tutorial.html

2 https://www.drive5.com/usearch/manual/uclust_algo.html

3 http://greengenes.secondgenome.com/

4 https://www.geneious.com/

5 https://www.rproject.org/

6 https://www.r-project.org/

## Supplementary Material

FIGURE S1 Venn Diagram illustrating the unique and shared OTUs in rhizobacterial community from *Cistanthe lingiscapa* during PF (pre-flowering) and FF (full-flowering) during 2017 Flowering desert event.
