# Supplementary figures and images for: Composition, Predicted Functions and Co-occurrence Networks of Rhizobacterial Communities Impacting Flowering Desert Events in the Atacama Desert, Chile
Source: Front Microbiol. 2020 Apr 8;11:571. doi: 10.3389/fmicb.2020.00571 (PMC7156552; doi:10.3389/fmicb.2020.00571)

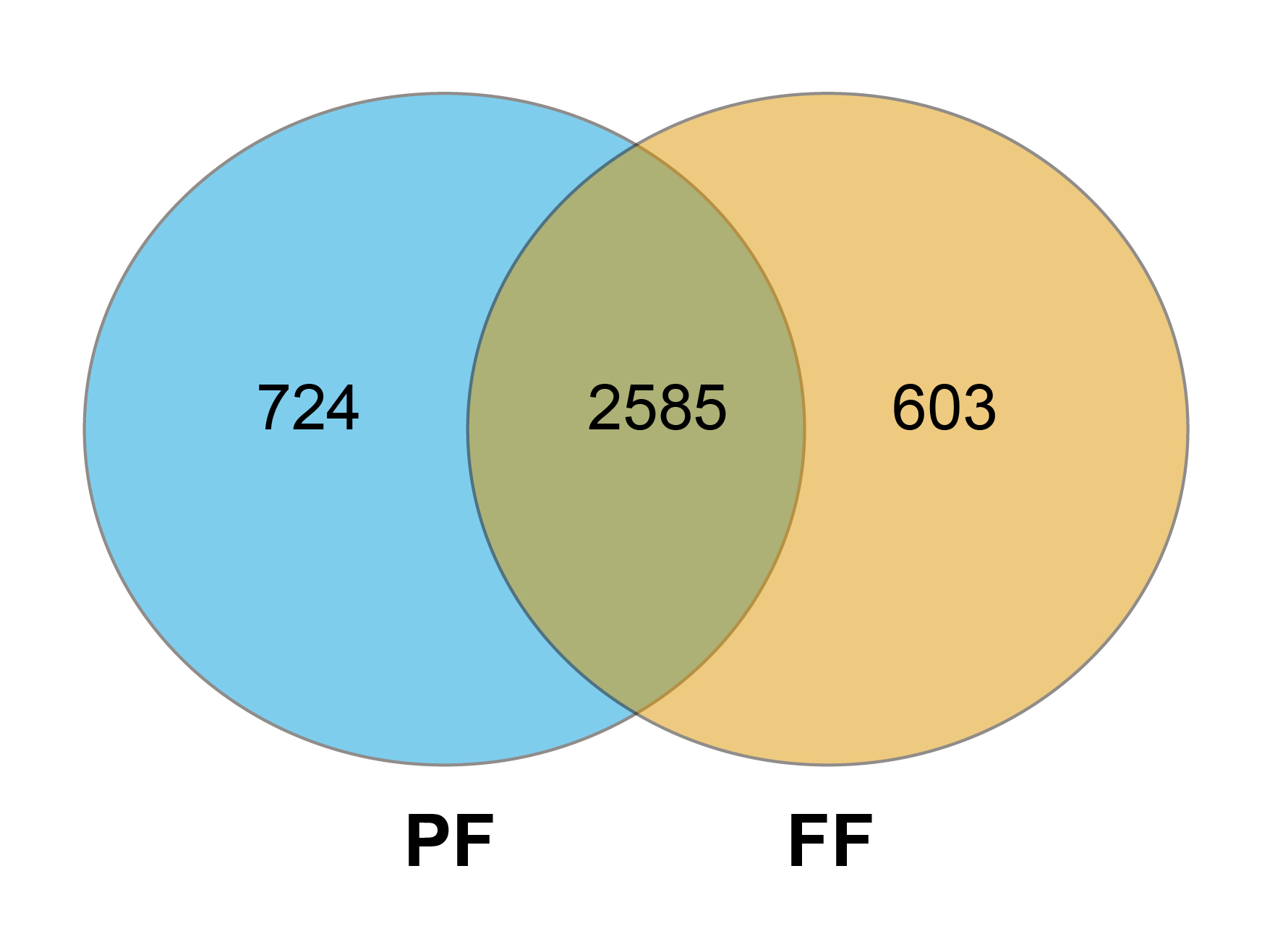

Supplement: FIGURE S1 — Venn Diagram illustrating the unique and shared OTUs in rhizobacterial community from Cistanthe lingiscapa during PF (pre-flowering) and FF (full-flowering) during 2017 Flowering desert event. [file Image_1.jpg]
